# Untargeted serum metabolomics reveals specific metabolite abnormalities in patients with Crohn's disease

**DOI:** 10.3389/fmed.2022.814839

**Published:** 2022-09-08

**Authors:** Huanhuan Liu, Minmin Xu, Qiongzi He, Peng Wei, Mengying Ke, Shijia Liu

**Affiliations:** ^1^Department of Pharmacy, Affiliated Hospital of Nanjing University of Chinese Medicine, Nanjing, China; ^2^College of Pharmacy, Jiangsu Collaborative Innovation Center of Chinese Medicinal Resources Industrialization, Nanjing University of Chinese Medicine, Nanjing, China

**Keywords:** serum metabolomics, Crohn's disease, biomarker, LC-MS, metabolite profile

## Abstract

Crohn's disease (CD) is a subtype of inflammatory bowel disease (IBD) characterized by skip intestinal lesions that can occur in any part of the gastrointestinal tract. Currently, the diagnosis of CD is based on clinical history, physical examination and complementary diagnostic tests. It is challenging for physicians to make a definitive diagnosis. This study aimed to analyze the variation in metabolites in CD serum and identify potential predictive biomarkers of CD diagnosis. We collected serum samples from 316 subjects, including patients with CD and healthy controls (HCs). Serum metabolomics was conducted using liquid chromatography coupled to mass spectrometry. Potential biomarkers were screened and evaluated by univariate and multivariate analyses. A panel of two metabolites (deoxycholic acid and palmitic amide) was identified as a specific biomarker of CD. Receiver operating characteristic analysis (ROC) showed that the panel had a sensitivity of 80.25% with a specificity of 95.54% in discriminating CD patients from healthy controls. The biomarkers identified are increased in CD compared with healthy controls. Our approach successfully identified serum biomarkers associated with CD patients. The potential biomarkers indicated that CD metabolic disturbance might be associated with bile acid biosynthesis, fatty acids and energy metabolism.

## Introduction

Crohn's disease (CD), a subtype of inflammatory bowel disease (IBD), is characterized by skip intestinal lesions that can occur in any part of the gastrointestinal tract (1). CD usually typically presents with chronic, relapsing, progressive and destructive transmural inflammation. Patients with CD usually present with chronic abdominal pain, diarrhea, obstruction and/or perianal lesions (1).

The precise etiology and pathogenesis of CD remain poorly understood. The current understanding mainly involves environmental factors in a genetically susceptible host, genetic factors, a defective host mucosal immune system, and gut microbial dysbiosis (2). Mucosal inflammation is a consequence of a multifaceted interaction. Currently, the diagnosis of CD is mainly based on clinical history, physical examination and complementary diagnostic tests, including assays for serological and fecal biomarkers, cross-sectional and endoscopic imaging, and histological evaluation of biopsy specimens (3, 4). In addition, quantitative magnetic resonance imaging (MRI) biomarkers combined with magnetic resonance enterography (MRE) qualitative assessment has also been applied to the diagnosis of CD (5). However, there are potential drawback in the addition of quantitative sequences to MRE examinations including increased scan time and the need for further validation before use in therapeutic drug trials and clinical trials. Thus, it is challenging for physicians to make a definitive diagnosis. Identification of biomarkers to discriminate CD patients from healthy individuals and other IBDs patients is highly desirable.

Metabolomics, which refers to an analytical study with high-throughput profiling of metabolites with the size of <1,500 Da including biofluids, cells and tissues, can revealed high-abundance molecules in various states such as disease and treatment states (6). Therefore, metabolomics has been widely used for the early screening of metabolic biomarkers in numerous diseases as well as providing new insights into the pathophysiology of diseases (7–9). Currently, there are several approaches that are applied to metabolomics, such as nuclear magnetic resonance (NMR) spectroscopy (10), quantitative NMR (11) gas chromatography-mass spectrometry (GC-MS) (12), liquid chromatography-mass spectrometry (LC-MS) (13), and capillary electrophoresis mass spectrometry (CE-MS) (14). In the last few years, the metabolomic approach has been used to identify metabolites in breath (15), fecal (16), serum (17), and urine (18) samples to discriminate inflammatory disease patients from healthy volunteers. However, there are few reports on CD serum metabolomics to distinguish patients with CD from healthy individuals. Therefore, there is a need to perform more studies on patients with CD.

The primary aim of this study was to identify serum metabolite profiles that could be used to differentiate CD patients from healthy controls (HCs) and to identify predictive potential biomarkers. We also aimed to investigate whether metabolomics could provide new insight into the complex pathophysiology of CD.

## Materials and methods

### Participants

This cross-sectional study examined adult CD patients and HCs. One hundred and eight CD patients were Asian inpatients from the Affiliated Hospital of Nanjing University of Traditional Chinese Medicine from September 2017 to April 2019. At the same time, 158 healthy volunteers came from the physical examination center of the Affiliated Hospital of Nanjing University of Chinese Medicine as a healthy control group. The diagnosis of CD was confirmed by previously established clinical, radiological and endoscopic criteria as well as histological findings (2). Disease activity was assessed using the simplified CD activity index (CDAI). Active disease was defined as a simplified CDAI of >4 for CD (19). The patients with other comorbidities that might affect metabolic characteristics, such as diabetes and cardiovascular diseases were excluded from the study. Patients who are taking oral hormones or other therapeutic drugs such as immunosuppressant's will also be excluded. A group of healthy adult volunteers (*n* = 158) matched for age, gender, and ethnicity served as controls. All participants gave informed consent, and the study was approved by the Institutional Review Board and the Ethics Committee of the First Affiliated Hospital of Nanjing University of Traditional Chinese Medicine (approval number, 2015NL-126-03) and complied with the principles of the Declaration of Helsinki.

### Sample preparation

The serum samples were stored at −80°C until analysis. Each serum sample had a volume of 45 μL. After centrifugation for 30 s, 135 μL of acetonitrile was added to precipitate the protein. The samples were vortexed for several seconds and then rested for 3 h. Then, the samples were centrifuged for 10 min at 13,000 rcf at 4°C, and 153 μL of supernatant was dried in a Speed Vac sample concentrator at 45°C for 2 h and re-dissolved in 120 μL of 50% acetonitrile solution. Eighty microliters were placed in the injection vial after centrifugation. At the same time, 10 μL of each serum sample was combined to form a quality control (QC) sample and was processed with the same procedure used for the experimental samples. During analysis of the samples, one quality control (QC) sample was run after every 10 injections.

### Metabolomic assays

The serum samples were assayed using an Agilent Technologies 1290 infinity liquid chromatograph coupled with an AB Sciex 4600 TripleTOF (AB Sciex, Framingham, MA, USA). For the detection of metabolites, 3 μL aliquots of sample solution maintained at 4°C in an autosampler were injected onto a reversed-phase ACQUITY UPLC HSS T3 C_18_ column (100 × 2.1 mm, 1.8 μm) maintained at 40°C. Mobile phase A was 1 ‰ formic acid in water, and mobile phase B was acetonitrile. The flow rate was 0.4 mL/min, and the gradient elution program was as follows: 0.5 min, 3% B; 0.5–1.5 min, 20% B; 1.5–6 min, 60% B; 6–9 min, 95% B; 9–12 min, 95% B; 12–12.1 min, 3% B; and 12.1–16 min, 3% B.

All MS experiments were performed in positive and negative ion modes using a heated electrospray ionization (ESI) source. The mass spectrum parameters were as follows: mass range for time-of-flight mass spectrometry (TOF-MS), 60–1,000 m/z; source temperature, 550°C; atomization gas pressure, 50 psi; auxiliary gas pressure, 50 psi; curtain gas pressure, 35 psi; ion scanning voltage, 5,500 V (positive ion mode); and mass range for MS/MS, 40–1,000 m/z.

### Data processing and statistical analysis

After obtaining the UPLC-MS chromatograms, the original data were derived by Analyst ^®^TF 1.7 Software (AB Sciex, USA), the abnormal peaks were removed by PeakView (v.2.0, AB SCIEX), and the peaks were aligned by Markview (version 1.2.1AB Sciex). Finally, a three-dimensional data table including the mass-to-charge ratio, retention time and peak area was obtained, and all peaks were corrected by QC samples.

In this study, there were two types of variables, continuous and categorical variables, which are presented as the mean ± SD and number (%), respectively. For continuous variables, Shapiro-Wilk tests were used to test the normality of the distribution. Student's *t*-test and Mann-Whitney U-test were used for normally and non-normally distributed data, respectively. For categorical variables, chi-square tests were applied. The correlation between the levels of metabolites and the severity of CD was performed using Spearman's rank correlation (Rs). These analyses were performed using SPSS 20.0 software (IBM, Armonk, NY, USA), and *P* < 0.05 was considered statistically significant.

For metabolomic analysis, we reduced the resulting matrix by replacing all the missing values with a small value. The data were normalized using logarithmic transformation and Pareto scaling in MetaboAnalyst 5.0 (https://www.metaboanalyst.ca/). Identified metabolites were subjected to further statistical analysis by univariate and multivariate statistical methods. For univariate statistical analysis, a non-parametric test was applied to measure the significance of each metabolite. The *P*-values for each metabolite in all comparisons were corrected by MetaboAnalyst 5.0 (https://www.metaboanalyst.ca/), in which the threshold was set as 0.05. Correction for multiple comparisons was performed by testing the false discovery rate, and the Q value was reported. Multivariate statistical analysis involving principal component analysis (PCA) and orthogonal partial least-squares discriminant analysis (OPLS-DA) was performed using SIMCA version 14.0 (Umetrics, Umea, Sweden). In the OPLS-DA model, the goodness of fit and predictive capacity was evaluated by the values of *R*^2^ and *Q*^2^. The model is deemed stable and reliable if the values are close to 1. Moreover, a permutation test was performed to assess the goodness of fit of the OPLS-DA model. The model was considered valid if all *Q*^2^ and *R*^2^ values to the left were lower than the original points to the right. The variable importance in the projection (VIP) value, which was calculated in the OPLS-DA model, indicated the contribution of each feature to the regression model. The higher the VIP scores were, the greater the contribution was. Metabolites with VIP scores above 1 and false discovery rates (Q values) <0.05 were selected as metabolite candidates that were the major contributors to discrimination between the two groups of participants. Through multivariate and univariable analysis, the metabolites are searched in the database including the Human Metabolome Database (http://www.hmdb.cn), METLIN (http://metlin.scripps.edu) and KEGG (http://www.kegg.com).

To explore the best combination of significantly altered metabolites, a binary logistic regression (BLR) model was built on the basis of the binary outcome of patients with CD and HCs as dependent variables. The forward stepwise regression method and the Wald test were used to select altered metabolites and assess significance in the BLR prediction model, respectively. Moreover, the area under the receiver operating characteristic (ROC) curve (AUC) as well as sensitivity and specificity values were calculated to identify the performance of logistical regression models. This method was used to discover the most important metabolites until there were no more significant predictors from the data in SPSS 20.0 software (IBM, Armonk, NY, USA).

## Results

### Basic characteristics of the participants

In this study, 221 subjects (106 with CD and 115 HCs) were allocated to the discovery set to evaluate biomarkers, and 95 subjects (52 with CD and 43 HCs) were allocated to the validation set to test candidate biomarkers. The clinical characteristics of the subjects are listed in Table 1. We recorded age, gender information of all volunteers, and recorded multiple clinical test indicators of patients with CD, including: C-reactive protein (CRP), erythrocyte sedimentation rate (ESR), platelet (PLT), white blood cell (WBC), hemoglobin (HGB), fecal calprotectin, albumin (ALB) and uric acid (UA) It can be seen that these representative indicators of CD patients are abnormally elevated.

**Table 1:** Clinical characteristics of the subjects.

**Characteristic**	**Discovery set** **(*****n*** = **221)**		**Validation set** **(*****n*** = **95)**	
	**CD**	**HC**	**CD**	**HC**
Number	106	115	52	43
Male	75	81	36	30
Female	31	34	16	13
Age (year)	28.6 ± 9.6	35.6 ± 10.8	26.3 ± 8.4	35.8 ± 9.7
CRP (mg/L)	12.1 ± 20.8	—	8.1 ± 12.5	—
ESR (mm/h)	19.3 ± 19.3	—	16.6 ± 16.4	—
PLT (10^9^/L)	253.4 ± 79.8	—	233.8 ± 103.3	—
WBC (10^9^/L)	6.1 ± 2.7	—	6.6 ± 3.6	—
HGB(g/L)	128.1 ± 21.7	—	126 ± 32	—
GWDB(μg/mL)	180.1 ± 347.3	—	249.9 ± 372.2	—
ALB(g/L)	36.1 ± 8.9	—	34 ± 12.8	—
UA(μmol/L)	295.1 ± 111.2	—	321.3 ± 139.8	—

Results are expressed as the means ± standard deviation.

CRP, C-reactive protein; ESR, erythrocyte sedimentation rate;PLT, platelet; WBC, white blood cell; HGB, hemoglobin; GWDB, Fecal Calprotectin; ALB, Albumin; UA, Uric acid.

### Identification of serum differential metabolites

**Figure 1:**
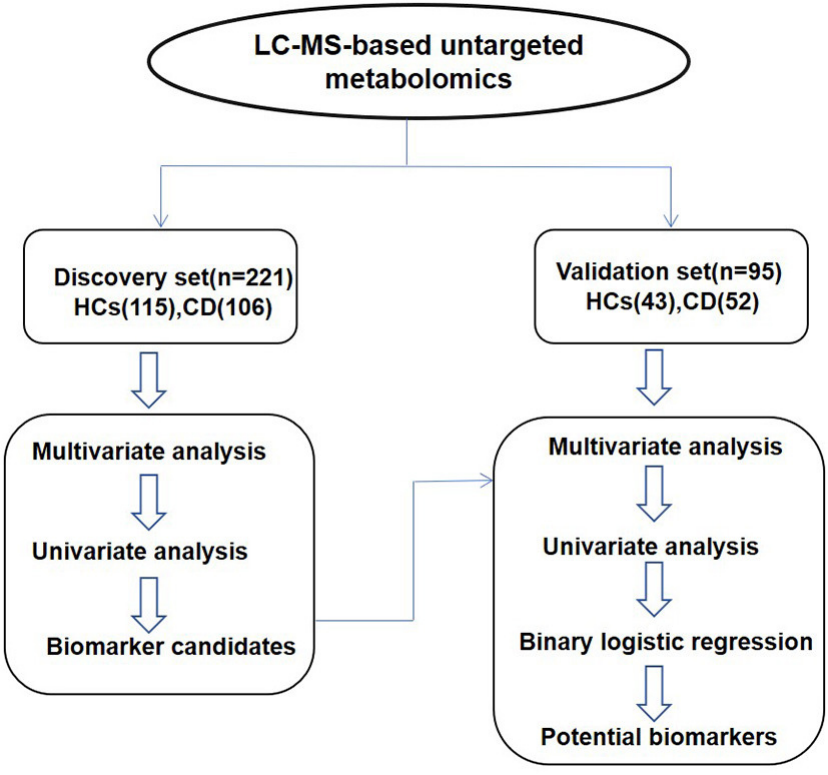
The whole workflow of this study.

**Figure 2:**
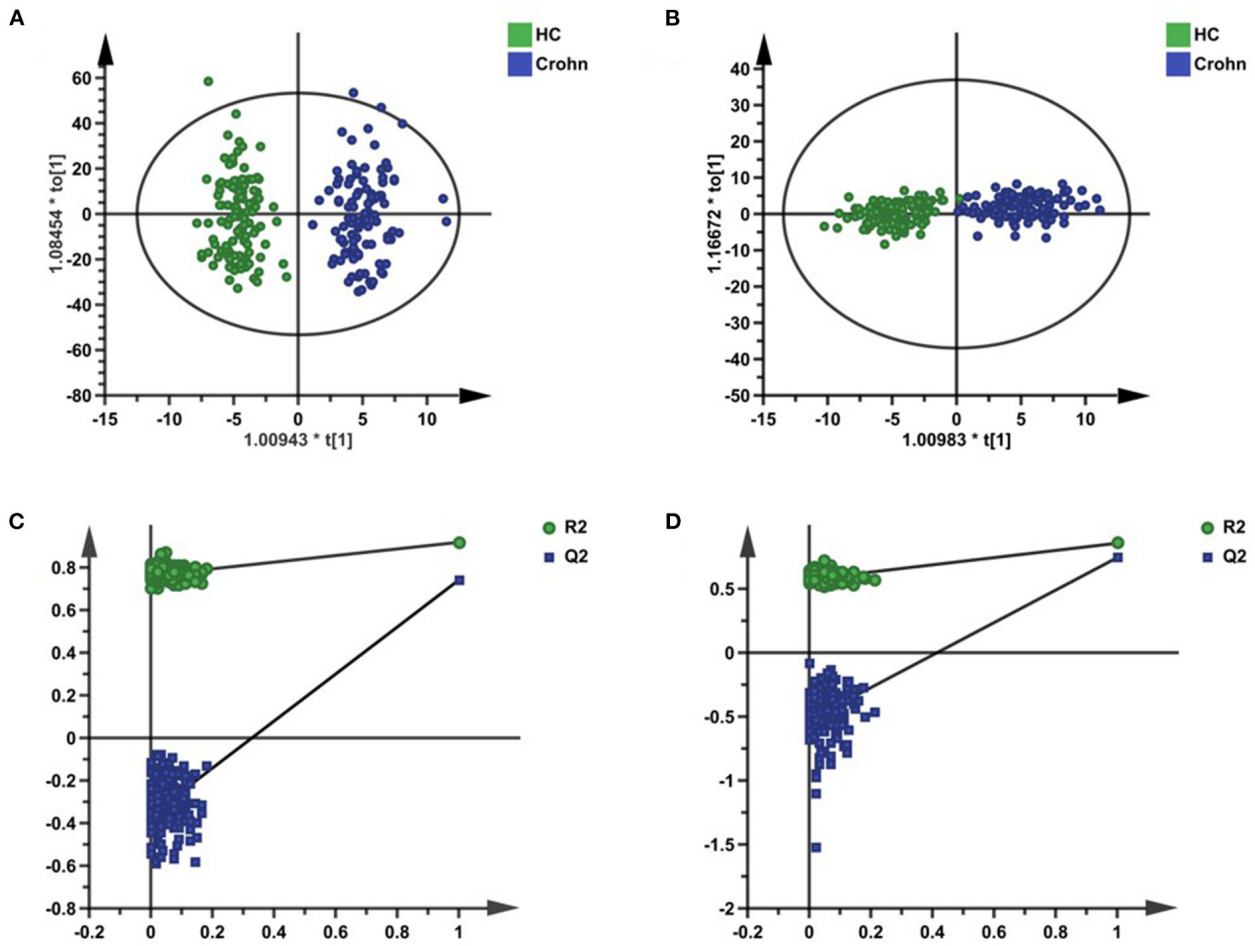
Multivariate statistical analysis of serum metabolites in the discovery set. **(A,B)** The OPLS-DA scatter plots were based on the serum metabolic profiles of CD patients and HCs in positive ion mode and negative ion mode. **(C,D)** The 200-time permutation plots of two OPLS-DA models.

**Figure 3:**
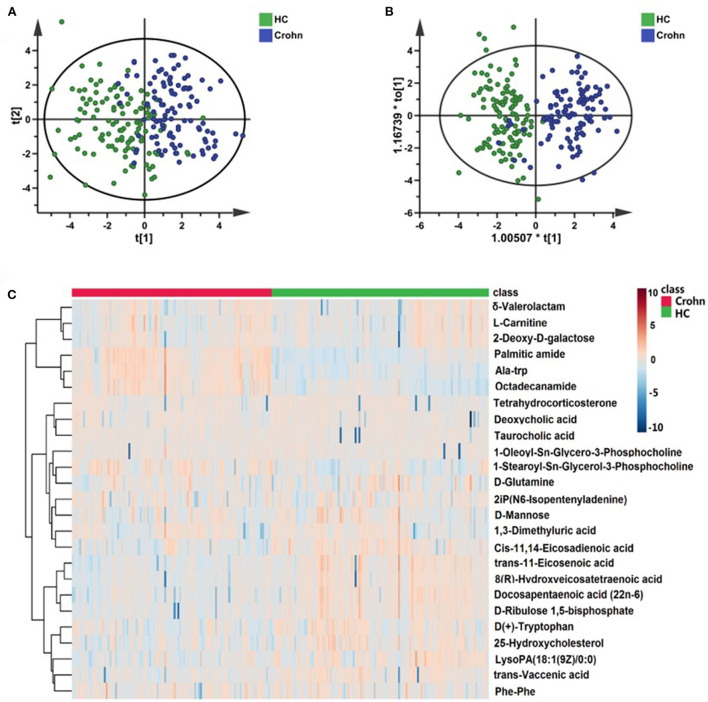
The performance of 25 differential metabolites for classification in the discovery set. **(A)** Heatmap of cluster analysis of each metabolite. **(B)** The scatter plot of PCA analysis was based on 25 metabolites. **(C)** The OPLS-DA scatter plot of 25 metabolites.

The whole workflow of this study is shown in Figure 1. A total of 1,690 features in positive mode and 1,021 features in negative mode were detected using UPLC-TOF-MS. After data processing, we conducted multivariate statistical analysis using SIMCA. The PCA could outline the original distribution of metabolites, but the scatter plot failed to show a clear distribution in both modes. As show in Figures 2A,B, the OPLS-DA scatter plot could be divided into two clusters, which indicated the differentiation between the groups. The models presented satisfactory fit (*R*^2^ = 0.917, *Q*^2^ = 0.741 in positive ion mode; *R*^2^ = 0.856, *Q*^2^ = 0.743 in negative ion mode). Furthermore, the 200-permutation test indicated all *Q*^2^ and *R*^2^ values to the left were lower than the original points to the right which results validated the OPLS-DA models (Figures 2C,D). Combined with univariate Wilcoxon rank-sum test (adjusted *P* < 0.05), we screened 25 differential metabolites (Supplementary Table S1). In addition, the 25 differential metabolites were subjected to PCA, OPLS-DA and cluster analysis. As show in Figure 3, both in the PCA, OPLS-DA, the samples were clearly separated between the two groups. Heatmap shows that these 25 metabolites are significantly different. Therefore, the 25 differential metabolites could well-distinguish CD patients from HCs.

### Validation of three potential biomarkers with a test set

An independent test cohort of 95 individuals (Figure 1) was used to evaluate the reliability of 25 biomarker candidates and confirm the application potential of the biomarkers. The OPLS-DA scatter plots (Supplementary Figures S1A,B) show the separation between the two groups, with good fitness (*R*^2^ = 0.921, *Q*^2^ = 0.641 in positive ion mode; *R*^2^ = 0.908, *Q*^2^ = 0.490 in negative ion mode), and both the models are validated (Supplementary Figures S1C,D). Five metabolites were verified in the validation cohort (Table 2). The table shows the *P*-value, FDR value, VIP value, and FC value of these five metabolites in the discovery set and validation set, respectively. These metabolites satisfied the following criteria: (1) VIP scores above 1 and false discovery rates (*Q*-values) below 0.05 and (2) maintaining the same change trend as the discovery set.

**Table 2:** Differentially altered metabolites identified between patients with CD and HCs.

**Metabolite**	**Discovery set**				**Validation set**			
	***P-*value**	**FDR**	**VIP**	**FC**	***P*-value**	**FDR**	**VIP**	**FC**
Deoxycholic acid	<0.001	<0.001	4.257	6.733	<0.001	<0.001	3.043	6.836
Docosapentaenoic acid (22n-6)	<0.001	<0.001	1.474	1.264	<0.001	<0.001	1.624	1.428
Octadecanamide	<0.001	<0.001	3.698	2.967	<0.001	<0.001	1.695	4.549
Palmitic amide	<0.001	<0.001	4.171	4.202	<0.001	<0.001	2.184	5.678
Tetrahydrocorticosterone	<0.001	<0.001	1.746	0.669	<0.001	<0.001	1.370	0.631

Five metabolites which were verified in the validation cohort. VIP was obtained from OPLS-DA model with a threshold of 1.0. P-values from one-way ANOVA. Value of FDR was obtained from the adjusted P-value calculated using MetaboAnalyst 5.0 software. FC was obtained by comparing those metabolites in patients with CD with the healthy controls; FC with a value >1 indicated a relatively higher intensity presenting in patients with CD, whereas a value <1 indicated a relatively lower intensity compared with the healthy controls.

**Figure 4:**
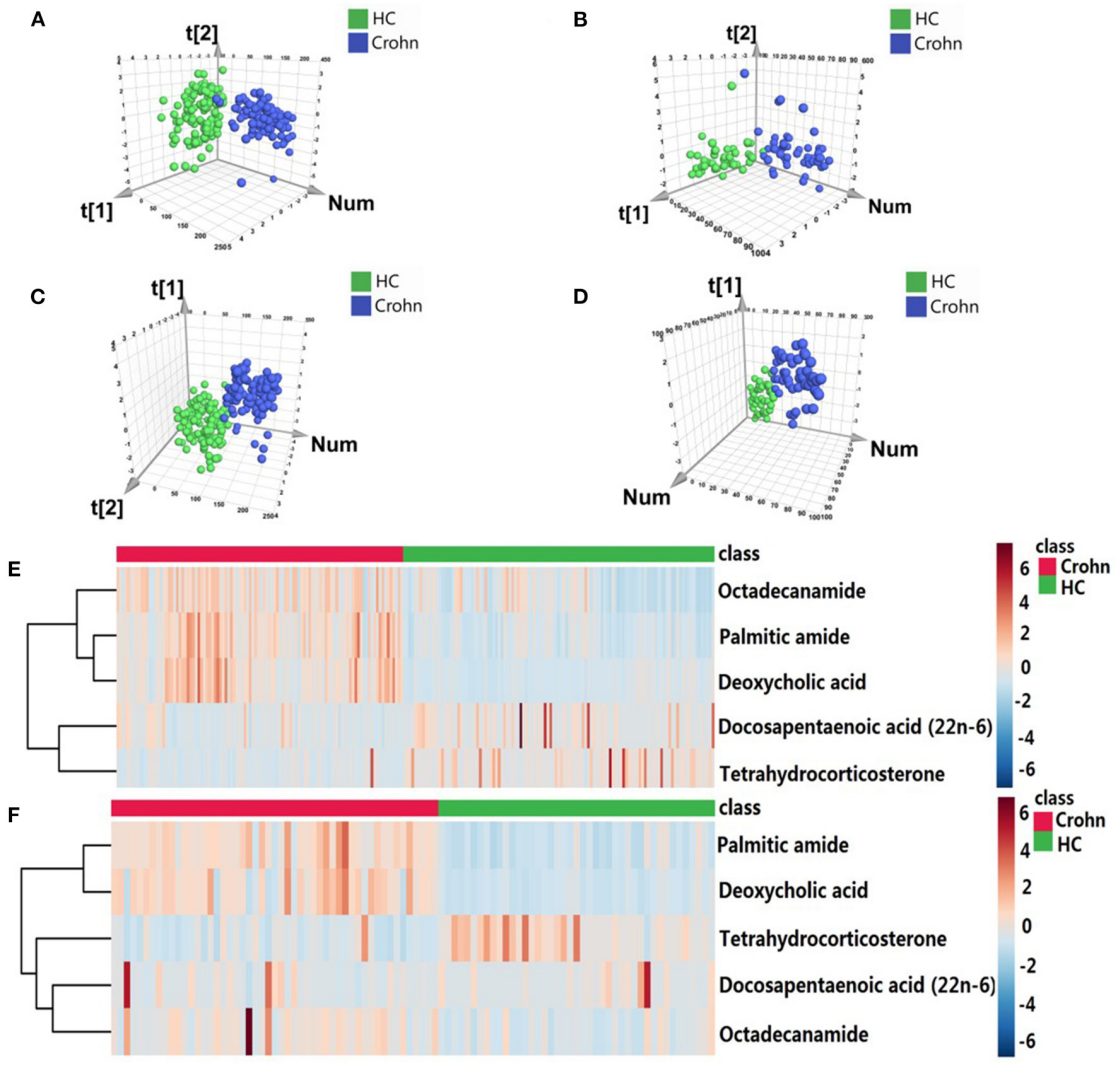
The performance of five overlapping metabolites for classification. The scatter plots of PCA based on five metabolites in the **(A)** discovery and **(B)** validation cohorts. The PLS-DA scatter plots of five metabolites in the **(C)** discovery and **(D)** validation cohorts. Heatmap of cluster analysis of each metabolite in the **(E)** discovery and **(F)** validation cohorts.

**Figure 5:**
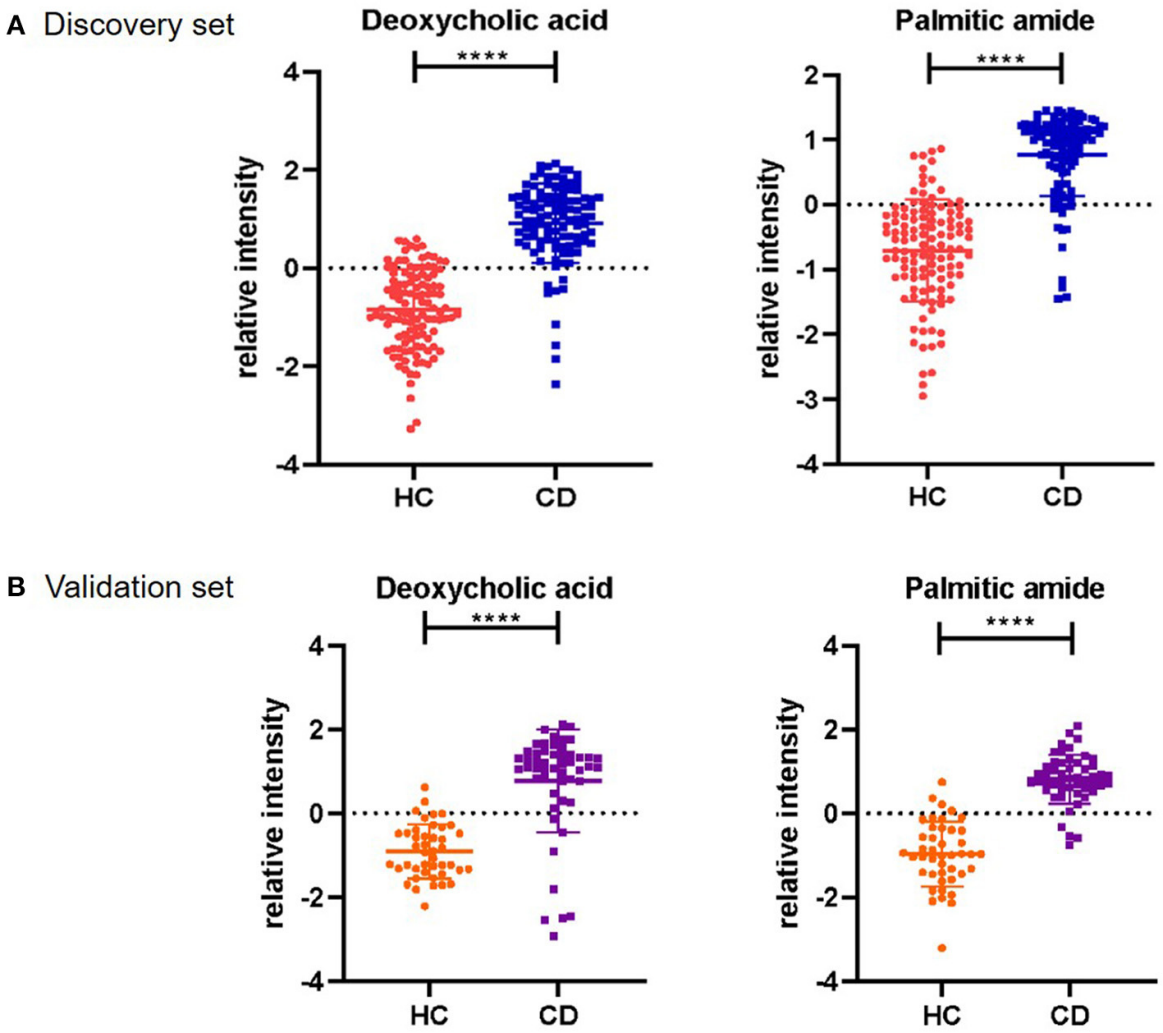
Serum relative intensity of the defined potential biomarkers. Discrimination of CD patients and healthy individuals with the combination of two potential biomarkers. Serum relative intensities of Deoxycholic acid and Palmitic amide in the discovery set **(A)** and validation set **(B)**. Statistical differences are marked by an asterisk, ****P < 0.0001.

**Figure 6:**
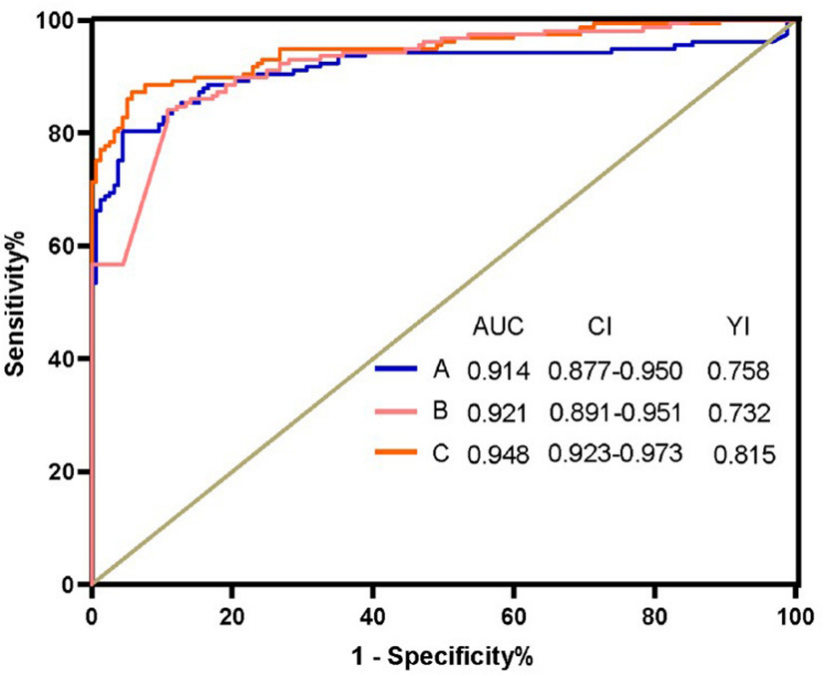
Receiver operating characteristic curve analysis (ROC) of Deoxycholic acid, Palmitic amide and their combination. (A) ROC of Deoxycholic acid (B) ROC of Palmitic amide. (C) ROC of the combination Deoxycholic acid, Palmitic amide. AUC, area under the curve; CI, confidence interval; YI, Youden index.

To construct the optimal diagnostic model, firstly, we detected the classification performance of the five metabolites. As shown in Figure 4, PCA, OPLS-DA and cluster analysis indicated that the five metabolites could separate CD patients from HCs. Additionally, we conducted BLR to optimize the model further. Through forward BLR analysis, two of the five metabolites remained in the logistics regression model: deoxycholic acid (DCA) and palmitic amide. Both the DCA and palmitic amide levels were significantly increased in patients with CD (Figure 5). The ROC values of the two metabolites and their combination are presented in Figure 6. For DCA, palmitic amide and their combination showed AUCs of 0.914, 0.921, and 0.948, sensitivities of 87.26, 84.08, and 80.25%, and specificities of 94.27, 89.17, and 95.54%, respectively.

**Figure 7:**
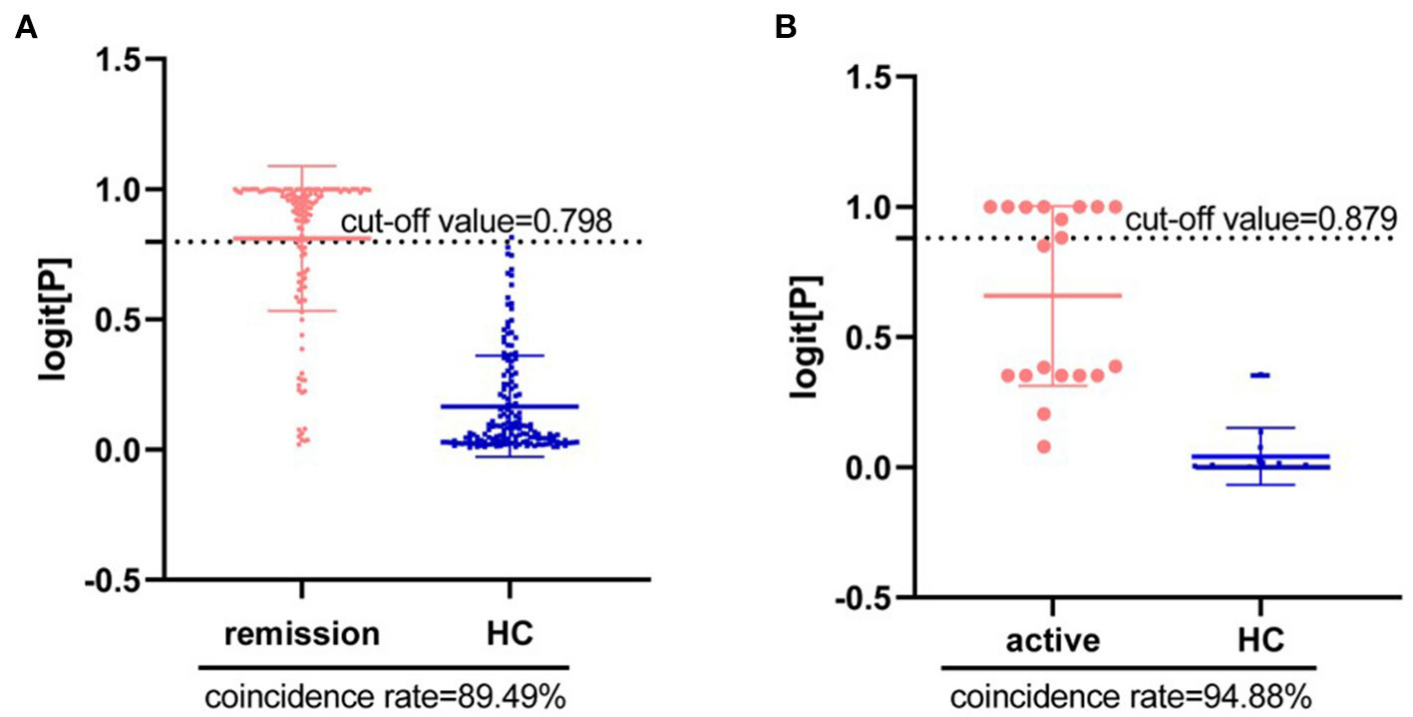
Diagnosis of disease activity in Crohn's Disease using Deoxycholic acid and Palmitic amide. **(A)** Diagnostic coincidence rate for the comparison between remission CD vs. HCs. **(B)** Diagnostic coincidence rate for the comparison between active CD vs. HCs. The vertical axis is the predicted probability. The horizontal axis represents the group.

Next, we tried to diagnose the disease activity of CD using DCA and palmitic amide. According to the latest CD staging standard, scores of <4, 5–8 and >9 are considered mild (or remission), moderate and severe disease activity, respectively. The results showed that there were significant differences in the levels of the two metabolites in different disease status of CD. As shown in Figures 7A,B, the combination of DCA and palmitic amide distinguishes HCs from remission CD and active CD, with a coincidence rate of 89.49 and 94.88%, and cut-off value of 0.798 and 0.879, respectively. The results indicate that the two differentially expressed metabolites could separate CD patients from HCs with high sensitivity, specificity and diagnostic performance. Finally, we confirmed the combination of DCA and palmitic amide as the ideal biomarker panel to distinguish patients with CD from HCs.

## Discussion

In this study, we demonstrated that there were significant differences in serum metabolic profiles between CD patients and HCs. We also confirmed the ideal biomarker panel to distinguish patients with CD from subjects without CD. The strengths of this paper are the relatively high number of cases and controls and the use of independent discovery and validation sets in building a discriminatory model. To the best of our knowledge, this is the first report on screening a specific biomarker panel to distinguish CD patients from HCs using untargeted serum metabolomics.

DCA is a secondary bile acid produced in the liver and is usually conjugated with glycine or taurine. This acid facilitates fat absorption and cholesterol excretion. DCA independently induces NLRP3 inflammasome activation and high proinflammatory cytokine-IL-1β production in macrophages (20). DCA triggers the activation of NLRP3 inflammasome by at least partially promoting the release of cathepsin B through sphingosine-1-phosphate receptor 2. In this study, compared with that in the healthy group, the DCA level in the CD group was significantly higher, by more than 6-fold. Thus, this result indicated that CD might be activate NLRP3 inflammasome induced by high levels of DCA, which could provide insights into the pathogenesis of CD in the future.

Palmitic amide is a primary fatty acid amide coming from palmitic acid (C16:0). Palmitic amide competes with other active substances such as cannabinoids or fatty acid amide hydrolase (degrading endocannabinoids), thereby increasing its concentration by preventing its degradation (21). William (22) et al. found that the activation of cannabinoid receptors both on immune cells and colonocytes is essential to prevent colitis and can be used as a preventive and treatment method for colitis. In our study, the concentration of palmitic amide in the CD group was significantly higher than that in the healthy group; thus, we proposed that the activation of cannabinoid receptors in CD patients was blocked.

In a recent publication, Lai et al. (23) reported that there was a unique metabolic pattern in patients with CD compared to that in HCs, and the identified differential compounds were structurally diverse, pointing to important pathway perturbations ranging from energy metabolism (e.g., β-oxidation of fatty acids) to signaling cascades of lipids (e.g., DHA) and amino acids (e.g., L-tryptophan). In our study, most of the metabolic pathway changes were also in lipid, amino acid and energy metabolism; in addition, disturbances in steroidogenesis were discovered. The metabolites of tetrahydrocorticosterone were significantly decreased in patients with CD. Tetrahydrocorticosterone is one of the major metabolites of corticosterone. Glucocorticoids are steroid hormones that decrease the severity of IBD by suppressing the immune response. In a previous study, Huang et al. (24) showed that during chronic intestinal inflammation, intestinal glucocorticoid synthesis was inhibited. In our study, CD patient steroid synthesis was blocked, and glucocorticoids were downregulated, which was consistent with previous studies.

Previous studies have demonstrated abnormal lipid and bile acid metabolism in patients with CD. In patients with CD, the terminal ileum is damaged, leading to malabsorption of bile acids and lipids, which in turn leads to diarrhea. Ineffective absorption of n-3 polyunsaturated fatty acids (PUFAs) reduces its anti-inflammatory effect (25). In our study, docosapentaenoic acid which is one of the n-3 PUFAs was significantly elevated in CD patients, indicating that its absorption was significantly reduced and thus the anti-inflammatory effect was reduced and the disease occurred. In addition, 7α-hydroxy-4-cholesten-3-one (C4) is a stable bile acid precursor. Robert et al. (26) observed significantly increased serum concentrations of C4 in patients with CD and indicated C4 may be a biomarker to identify patients with diarrhea attributable to bile acid malabsorption. Vitamin D regulates the immune system by reducing Th1/Th17 T cells, inflammatory cytokines, etc, and vitamin D absorption is significantly reduced in patients with CD. Therefore, abnormal metabolism of vitamin D also perturbs lipid metabolism in CD patients.

There are more research about biomarkers in the field of CD. In a value review, Mohsen et al. (27) address CD biomarkers including serologic biomarkers, genetic predisposing markers and Interleukin-24. However, their specificity and accuracy need to be further improved, for example, CRP and ESR, which are cheap and reliable, but hugely non-specific. At the end of the article the author mentioned the need for more novel biomarkers like metabolomics technology in the future, leading to highly accurate testing. In our study, an LC-MS/MS metabolomic method was applied to compare the serum metabolic characteristics between CD patients and HCs and a panel of two metabolites (DCA and palmitic amide) was identified as a specific biomarker of CD.

In recent years, research on fecal metabolites and IBD is rapidly increasing and improving with the development of technology. However, the characterization of the human fecal metabolome still lags behind these other metabolomes because of various reasons, such as standardized methods and freely available resources (28). In a past review study on colorectal cancer, we found that serum and tissue are the preferred biological samples, and the analysis of stool samples only accounts for a small part (29). Since the acquisition of human intestinal tissue samples is invasive, the acquisition of serum samples is very convenient, and serum metabolomics is a classic metabolomics study. Therefore, we choose serum for metabolomics analysis.

According to the lesion site, CD can be divided into L1, terminal ileum; L2, colon; L3, ileocolon and L4, upper gastrointestinal tract. In our study, there is L1, L2, L3, L1L4, L2L4, and L3L4. The result of association between regio specific CD sites and markers is showed in Supplementary Table S2. It can be seen from the results that there is not significant association between regio specific CD sites and markers. It may be caused by the distribution of different types of samples or other reasons. In future research, we should pay attention to the selection of sample types and other factors that may affect the results. We have also conducted correlation analysis between clinical characteristics and biomarker (Supplementary Table S3). Because the results of the correlation analysis are not good, we do not add in the text. It may be due to the limitation of sample size. In the future research, we will try to expand the number of samples to design experiments.

There were three limitations in this study. Firstly, there were all CD patients from one center in our study. In the future, a large cohort of multicenter participants will be required to verify the reliability of the results. In this study, we are limited to cross-sectional studies to screen biomarkers, and further studies provide more longitudinal data to prove the role of these biomarkers in early diagnosis, monitoring disease evolution or time to relapse of the patient. Secondly, all patients were from Asia. We should include CD patients of different races for research in future studies. Lastly, we know that IBD mainly includes two subtypes, UC and CD. In our study, only CD patient samples were included. Therefore, performing further studies focused on metabolic profiling using UC patient samples are required. So, we can try to include UC patients to identify the metabolite profile that could be used to differentiate CD and UC patients, it will be a more challenging study.

## Conclusion

In conclusion, an LC-MS/MS metabolomic method was applied to compare the serum metabolic characteristics between CD patients and HCs in this study. A panel of two metabolites (DCA and palmitic amide), which were both upregulated, was identified as a specific biomarker of CD. These serum metabolites are mainly related to bile acid biosynthesis, fatty acids and energy metabolism. Our findings will provide a new method for the diagnosis of CD and new insights into CD pathogenesis.

## Data availability statement

The raw data supporting the conclusions of this article will be made available by the authors, without undue reservation.

## Ethics statement

The studies involving human participants were reviewed and approved by the Ethics Committee of the First Affiliated Hospital of Nanjing University of Traditional Chinese Medicine. The patients/participants provided their written informed consent to participate in this study.

## Author contributions

HL, MX, and SL were responsible for the conception and design of the study. QH performed the study retrieval. PW and MK collected samples. HL and MX contributed to the data collection and statistical analysis. HL drafted the manuscript. MX and SL were responsible for the revision of the manuscript. All authors contributed to the article and approved the submitted version.

## Funding

This work was financially supported by the National Natural Science Foundation of China (Nos. 82074241 and 81774096) and Jiangsu Provincial Department of Education (No. SJCX21_0693).

## Conflict of interest

The authors declare that the research was conducted in the absence of any commercial or financial relationships that could be construed as a potential conflict of interest.

## Publisher's note

All claims expressed in this article are solely those of the authors and do not necessarily represent those of their affiliated organizations, or those of the publisher, the editors and the reviewers. Any product that may be evaluated in this article, or claim that may be made by its manufacturer, is not guaranteed or endorsed by the publisher.
