# Supplementary material for: Untargeted serum metabolomics reveals specific metabolite abnormalities in patients with Crohn's disease
Source: Front Med (Lausanne). 2022 Sep 8;9:814839. doi: 10.3389/fmed.2022.814839 (PMC9492954; doi:10.3389/fmed.2022.814839)
Supplement: Supplementary file 1 [file Data_Sheet_1.pdf]

Supplementary Material

Supplementary Table S1. Serum Differential metabolites in Patients with CD Compared with Healthy Controls in the Discovery Set

metabolite name	VIP	Fold change	p.value	FDR	Average Rt(min)	Average Mz
Ala-trp	1.244	0.564	0.001	0.016	1.64	276.14342
2iP(N6-Isopentenyladenine)	1.072	0.647	0.001	0.004	1.68	202.10916
Tetrahydrocorticosterone	1.746	0.669	<0.001	<0.001	4.15	315.23487
25-Hydroxycholesterol	1.260	0.770	<0.001	<0.001	10.99	447.34816
D-Glutamine	1.312	0.828	<0.001	<0.001	0.76	145.06266
L-Carnitine	1.109	0.849	0.002	0.025	0.77	162.11177
1,3-Dimethyluric acid	1.394	0.853	<0.001	<0.001	0.77	217.03019
D-Mannose	1.469	0.854	<0.001	<0.001	0.77	215.03334
D-Ribulose 1,5-bisphosphate	1.234	0.894	0.005	0.018	0.78	330.94954
Phe-Phe	1.212	1.176	0.001	0.004	5.57	623.29201
LysoPA(18:1(9Z)/0:0)	1.448	1.196	<0.001	<0.001	7.20	457.23604
δ-Valerolactam	1.254	1.224	0.001	0.017	17.93	100.07556
2-Deoxy-D-galactose	1.465	1.250	0.003	0.041	0.82	187.05712
D (+)-Tryptophan	1.244	1.261	0.004	0.016	11.28	407.18916
Docosapentaenoic acid (22n-6)	1.474	1.264	<0.001	<0.001	4.63	329.23304
Taurocholic acid	1.393	1.266	0.002	0.031	5.54	516.30509
1-Stearoyl-Sn-Glycerol-3-Phosphocholine	1.737	1.267	<0.001	<0.001	6.52	524.36137
1-Oleoyl-Sn-Glycero-3-Phosphocholine	1.760	1.274	<0.001	0.003	6.52	522.35657
trans-Vaccenic acid	1.317	1.343	<0.001	<0.001	10.94	563.50435
Cis-11,14-Eicosadienoic acid	1.279	1.387	<0.001	0.003	11.33	307.26420
trans-11-Eicosenoic acid	1.103	1.421	0.005	0.019	12.41	309.27974
8(R)-Hydroxy-(5Z,9E,11Z,14Z)-eicosatetraenoic acid	2.195	2.508	<0.001	<0.001	6.84	319.22757
Octadecanamide	3.698	2.967	<0.001	<0.001	13.05	284.29448
Palmitic amide	4.171	4.202	<0.001	<0.001	11.43	256.26333
Deoxycholic acid	4.257	6.733	<0.001	<0.001	5.24	393.28543

VIP was obtained from OPLS-DA model with a threshold of 1.0. P values from one-way ANOVA. Value of FDR was obtained from the adjusted P value calculated using MetaboAnalyst 5.0 software. FC was obtained by comparing those metabolites in patients with CD with the healthy controls; FC with a value >1 indicated a relatively higher intensity presenting in patients with CD, whereas a value <1 indicated a relatively lower intensity compared with the healthy controls.

Supplementary Table S2. The association between regio specific CD sites and markers

Biomarker	L1vsL2	L1vsL3	L2vsL3	L1vsL4-M	L2vsL4-M	L3vsL4-M
deoxycholic acid	0.3125	0.6431	0.5802	0.7207	0.6540	0.8209
palmitic amide	0.2822	0.7081	0.3188	0.8604	0.3097	0.7300

P value between regio specific CD sites and markers obtained from Mann-Whitney U test.

L4-M containing L1L4, L2L4 and L3L4.

Supplementary Table S3. The correlation between clinical characteristics and biomarker

Biomarker	CRP	ESR	PLT	WBC	HGB	GWDB	PA	ALB	UA
deoxycholic acid	0.018	-0.003	0.055	-0.034	-0.098	-0.125	-0.098	-0.011	-0.024
palmitic amide	0.013	0.004	-0.082	-0.068	-0.080	0.083	-0.116	-0.005	0.013

The correlation analysis obtained by Spearman r.

CRP: C-reactive protein, ESR: erythrocyte sedimentation rate, PLT: platelet, WBC: white blood cell, HGB: hemoglobin, GWDB: Fecal Calprotectin, PA: prealbumin, ALB: Albumin, UA: Uric acid.

Fig. S1 Multivariate statistical analysis of serum metabolites in the validation set

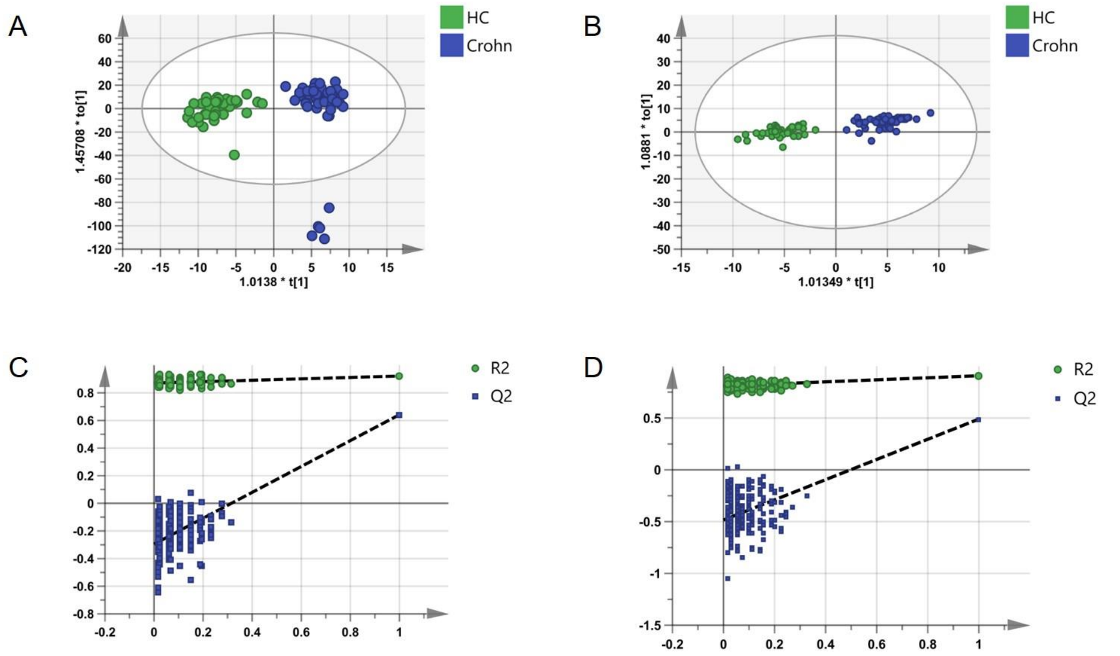

The OPLS-DA scatter plots were based on the serum metabolic profiles of CD patients and HCs in positive ion mode and negative ion mode. (C, D) The 200-time permutation plots of two OPLS-DA models.
